# CTBP1 depletion on prostate tumors deregulates miRNA/mRNA expression and impairs cancer progression in metabolic syndrome mice

**DOI:** 10.1038/s41419-019-1535-z

**Published:** 2019-04-01

**Authors:** Guillermo Nicolás Dalton, Cintia Massillo, Georgina Daniela Scalise, Rocío Duca, Juliana Porretti, Paula Lucia Farré, Kevin Gardner, Alejandra Paez, Geraldine Gueron, Paola De Luca, Adriana De Siervi

**Affiliations:** 1 Laboratorio de Oncología Molecular y Nuevos Blancos Terapéuticos, Instituto de Biología y Medicina Experimental (IBYME), CONICET, Argentina; 2 Department of Pathology and Cell Biology, Columbia University Medical Center, 630 W. 168th Street, New York, NY 10032 USA; 3 Departamento de Química Biológica, Universidad de Buenos Aires, Facultad de Ciencias Exactas y Naturales, Laboratorio de inflamación y Cáncer, Buenos Aires, Argentina

## Abstract

About 20% of prostate cancer (PCa) patients progress to metastatic disease. Metabolic syndrome (MeS) is a pathophysiological disorder that increases PCa risk and aggressiveness. C-terminal binding protein (CTBP1) is a transcriptional corepressor that is activated by high-fat diet (HFD). Previously, our group established a MeS/PCa mice model that identified CTBP1 as a novel link associating both diseases. Here, we integrated in vitro (prostate tumor cell lines) and in vivo (MeS/PCa NSG mice) models with molecular and cell biology techniques to investigate MeS/CTBP1 impact over PCa progression, particularly over cell adhesion, mRNA/miRNA expression and PCa spontaneous metastasis development. We found that CTBP1/MeS regulated expression of genes relevant to cell adhesion and PCa progression, such as cadherins, integrins, connexins, and miRNAs in PC3 xenografts. CTBP1 diminished PCa cell adhesion, membrane attachment to substrate and increased filopodia number by modulating gene expression to favor a mesenchymal phenotype. NSG mice fed with HFD and inoculated with CTBP1-depleted PC3 cells, showed a decreased number and size of lung metastases compared to control. Finally, CTBP1 and HFD reduce hsa-mir-30b-5p plasma levels in mice. This study uncovers for the first time the role of CTBP1/MeS in PCa progression and its molecular targets.

## Introduction

Prostate cancer (PCa) is the second most diagnosed cancer type and the fifth cause of death by cancer among males worldwide^1^. Most PCa-related deaths are due to advanced disease, which results from any combination of lymphatic, hematogenous, or contiguous local spread^2^. About 90% of patients in the final stages of PCa, known as castration resistant prostate cancer (CRPC), will develop bone metastases which dramatically reduce patient survival and quality of life^3^.

Metabolic syndrome (MeS) is one of the most widely prevailing health concerns worldwide. It is a cluster of pathophysiological disorders whose diagnose requires the detection of, at least, three of the following factors: visceral adiposity, high triglycerides, low-high density lipoprotein (HDL) cholesterol levels, high-blood pressure, and elevated fasting glucose levels^4^. Latest estimates indicate a worldwide prevalence ranging between 10 and 40%, depending on lifestyle and genetic background^5^. Diet, lifestyle, and genetic background not only affect MeS, there is an increasing body of evidence showing that these factors play a crucial role in PCa risk and progression^6–8^. Likewise a recent meta-analysis found a significant correlation associating MeS with more aggressive PCa tumors and biochemical recurrence^9^. Nonetheless, the molecular players responsible for the effect of MeS on the progression/aggressiveness of PCa tumors are yet to be completely identified.

Recent years have seen an overflow of reports regarding miRNAs role in cancer. Many reviews have been published on miRNAs deregulation in cancer, both as cause and consequence, and as possible biomarkers or therapeutic molecules^10–13^.

Previously our group identified C-terminal binding protein 1 (CTBP1) as a link between MeS and PCa^14,15^. CTBP1 is a transcriptional corepressor of many tumor suppressor genes. Binding either NAD+ or NADH is necessary for CTBP1 activation; however, CTBP1 affinity is 100-fold higher for NADH making it a molecular sensor of the metabolic state of the cell^16^. We previously generated a murine model of MeS and PCa by chronically feeding animals with high-fat diet (HFD). This model allowed us to identify novel pathways regulated by CTBP1 on a MeS environment^14^. CTBP1 depletion in prostate xenografts developed in MeS *nu/nu* mice dramatically decreased tumor growth and modulated cell adhesion, metabolic process, and cell cycle-related genes^14^. Moreover, we recently described a novel regulation of cell adhesion and epithelial-to-mesenchymal transition (EMT) in PCa cells by the repression of chloride channel accessory 2 (*CLCA2)* mediated by CTBP1 and miR-196b-5p. Also, we demonstrated that *CLCA2* is a target of miR-196b-5p^15^.

In this work our aim was to understand CTBP1 and related miRNAs role on PCa progression. We demonstrated that CTBP1 decreases the in vitro adhesive capabilities of a panel of PCa cell lines through the modulation of genes like Cadherin 1 (*CDH1)*, Integrin Subunit Beta 4 (*ITGB4)*, and Vimentin (*VIM)* among others. Consistently, CTBP1 favors a mesenchymal and pro-invasive phenotype. Using a MeS and spontaneous PC3 metastasis in vivo model, we found that CTBP1 depletion on MeS mice impairs the development of lung metastases. In addition, we show that CTBP1 regulates a cluster of miRNAs that target cell adhesion genes, which could in turn impact over cell adhesion itself and ultimately on the onset of metastatic disease.

## Results

### CTBP1 regulates expression of mRNAs and miRNAs involved in cell adhesion on a PC3 and MeS in vivo model

**Fig. 1:**
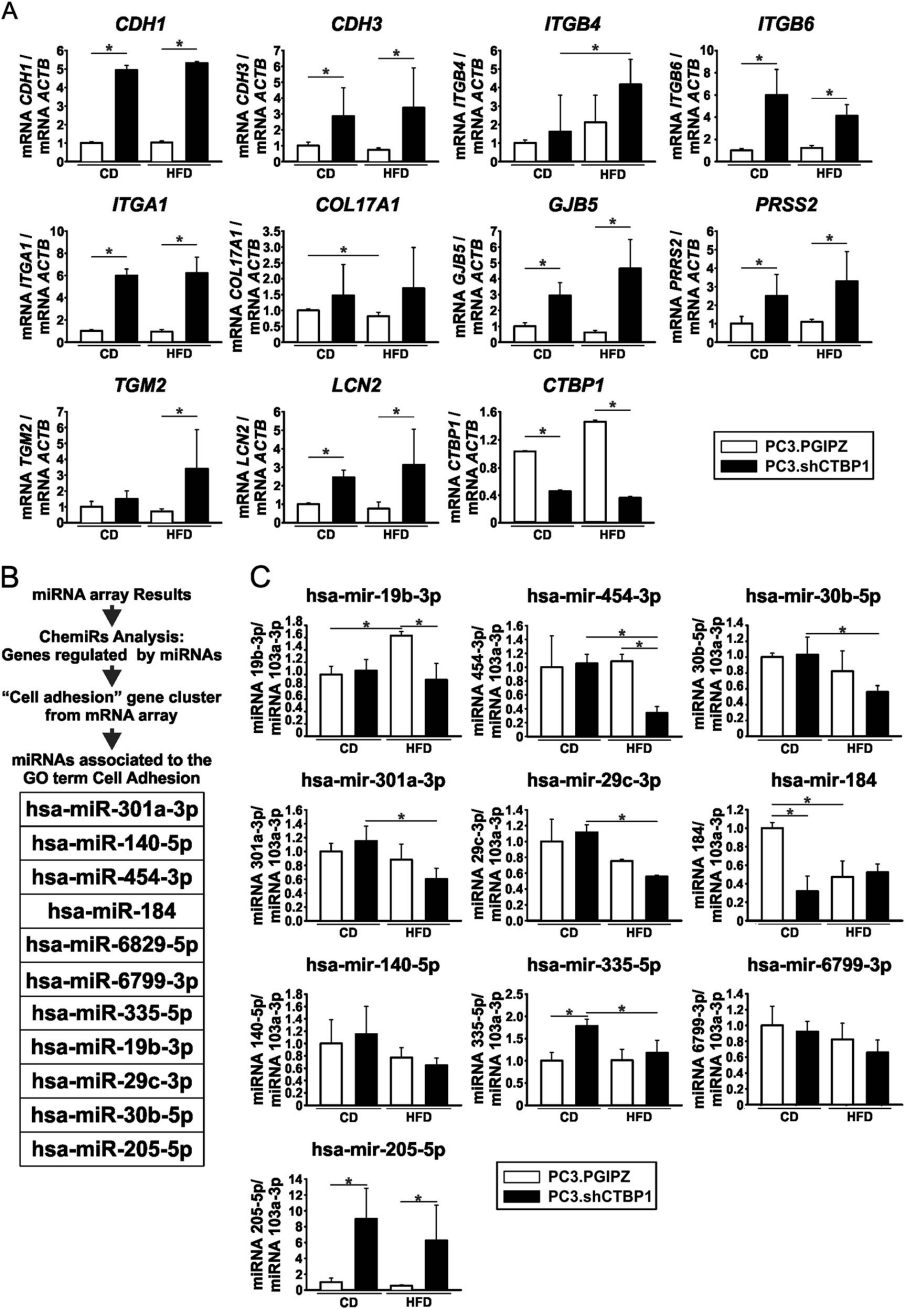
CTBP1 regulates mRNAs and miRNAs related to GO “cell adhesion” in xenografts grown on MeS mice. Expression of the indicated mRNAs (**a**) and miRNAs (**c**) in xenografts from CD or HFD *nu/nu* mice inoculated with PC3.shCTBP1 or PC3.PGIPZ cells were determined by RT-qPCR using specific primers. Data were normalized to *ACTB* and control for mRNAs or to hsa-miR-103a-3p and control for miRNAs. Bars represent the average and SD from three independent mice. Significance was analyzed by two-way ANOVA. ^*^p ≤ 0.05. **b** Flow chart and list of miRNAs associated to the GO term “cell adhesion”

We previously reported a mice model of PCa and MeS^14^. Briefly, male *nu/nu* mice fed with control diet (CD) or HFD during 12 weeks, were s.c. inoculated with CTBP1 depleted (PC3.shCTBP1) or control (PC3.PGIPZ) PC3 cells. RNA isolated from xenografts grown on MeS mice was used to hybridize a whole-genome expression microarray (Affymetrix). Gene set enrichment analysis (GSEA) indicated that CTBP1 regulates many pathways^14^, being “cell adhesion” one of the more robustly represented. We selected a list from this set that included cancer progression related genes and validated it by real-time quantitative polymerase chain reaction (RT-qPCR). As shown on Fig. 1a, CTBP1 depletion and/or MeS increased the expression of cadherins (*CDH1* and *CDH3*), integrins (*ITGB4*, *ITGB6*, and *ITGA1*), collagen type XVII alpha 1 (*COL17A1*), connexin (*GJB5*), serine protease (*PRSS2*), transglutaminase (*TGM2*), and lipocalin (*LCN2*).

We previously reported that CTBP1 regulates miRNAs^15,17,18^. Concisely, RNA samples obtained from the xenografts tumors described above were used to hybridize a miRNA expression microarray (Affymetrix)^15^. In this work, miRNA and mRNA microarray results were cross referenced. We used ChemiRs database to obtain lists of genes regulated by miRNAs on the array and compared them to the cell adhesion cluster from the mRNA array (Fig. 1b). miRNA expression was determined by miRNA RT-qPCR from CTBP1-depleted xenografts or control, grown in CD or HFD-fed mice (Fig. 1c). Interestingly, CTBP1 depletion downregulated miRNAs related to cell adhesion, such as hsa-miR-19b-3p and hsa-miR-454-3p. Other miRNAs, hsa-miR-30b-5p, hsa-miR-301a-3p, and hsa-miR-29c-3p were downregulated by MeS and, hsa-miR-184, hsa-miR-140-5p, hsa-miR-335-5p, and hsa-miR-6799-3p could not be validated by this method (Fig. 1c). Moreover, hsa-miR-205-5p, a known tumor suppressor miRNA, was highly upregulated in CTBP1-depleted tumors (Fig. 1c).

### CTBP1 diminishes cell adhesion by modulating gene expression

**Fig. 2:**
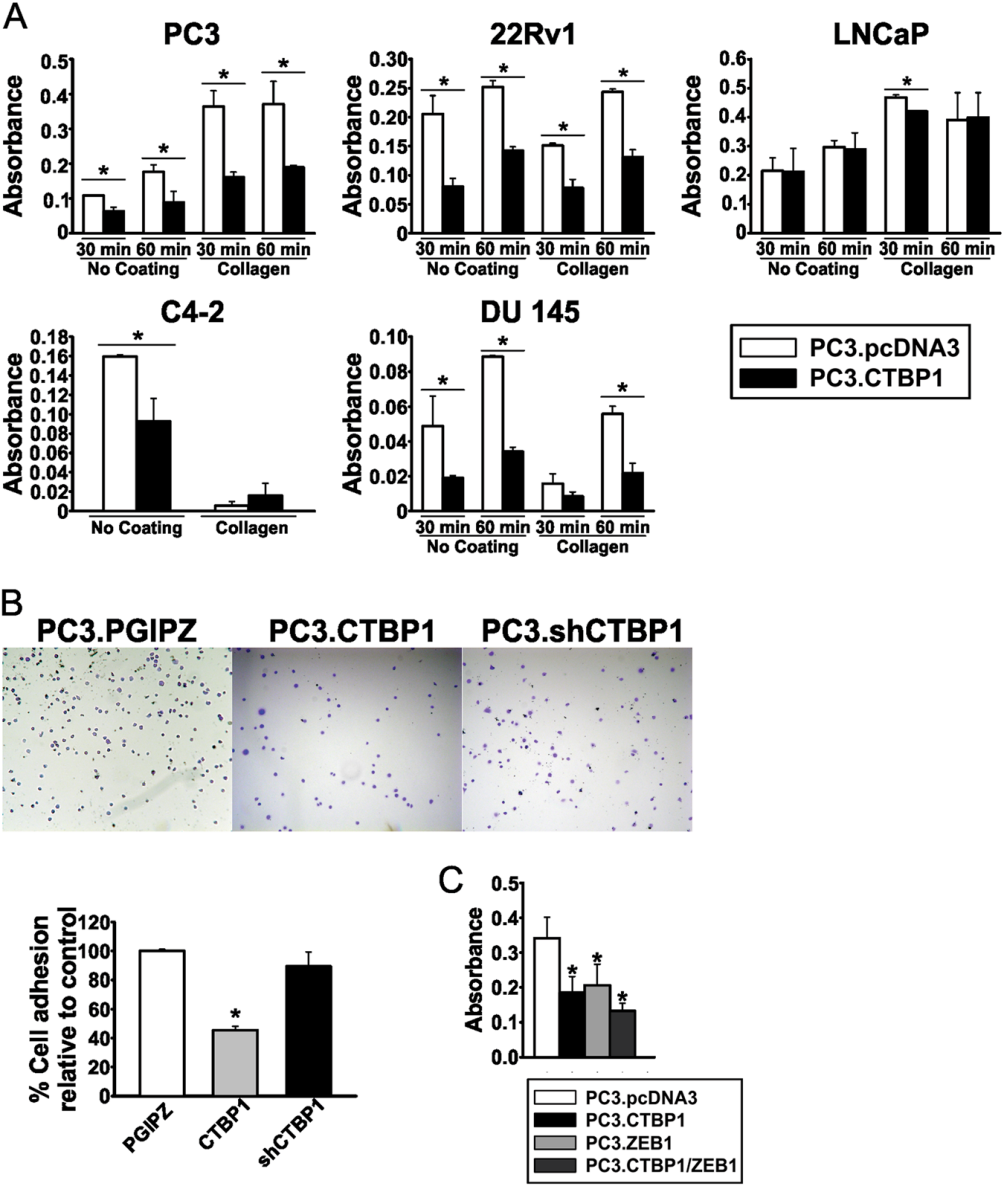
CTBP1 diminishes cell adhesion in PCa cell lines. Cell adhesion assay. Different PCa cell lines, with varying levels of CTBP1 expression were tested. **a** PC3, 22RV1, LNCaP, C4-2, and DU 145 cell lines transfected with a CTBP1 expression (PC3.CTBP1) or control (PC3.PCDNA3) plasmids. C4-2 cell line was tested only for 60 min. **b** PC3.shCTBP1 and its respective control PC3.PGIPZ. *Top*, a representative image of fixed cells stained with CV. *Bottom*, graphical representation of total amount of attached cells per treatment. **c** PC3 cells were transfected with a control, CTBP1 and/or ZEB1 expression plasmids. Adhesion was tested only for 60 min. In all cases bars represent the average and SD of a representative experiment. Data were analyzed by one-way ANOVA. *p ≤ 0.05

To explore the effect of CTBP1 on cell adhesion, we transfected PC3, 22Rv1, LNCaP, C4-2, and DU 145 cells with control (PC3.pcDNA3) or CTBP1 (PC3.CTBP1) expression plasmids, and assessed cell adhesion to collagen coated- or noncoated plastic. As shown in Fig. 2a, b, CTBP1 overexpression significantly diminished cell adhesion in all PCa cell lines under study.

It was previously reported that CTBP1 interacts with ZEB1 transcription factor to repress the expression of genes involved in EMT process, such as *CDH1*^19^. Hence, we investigated CTBP1 and/or ZEB1 overexpression effect over cell adhesion. Although, CTBP1 and ZEB1 overexpression decreased cell adhesion, no synergy was observed between these two coregulators (Fig. 2c).

**Fig. 3:**
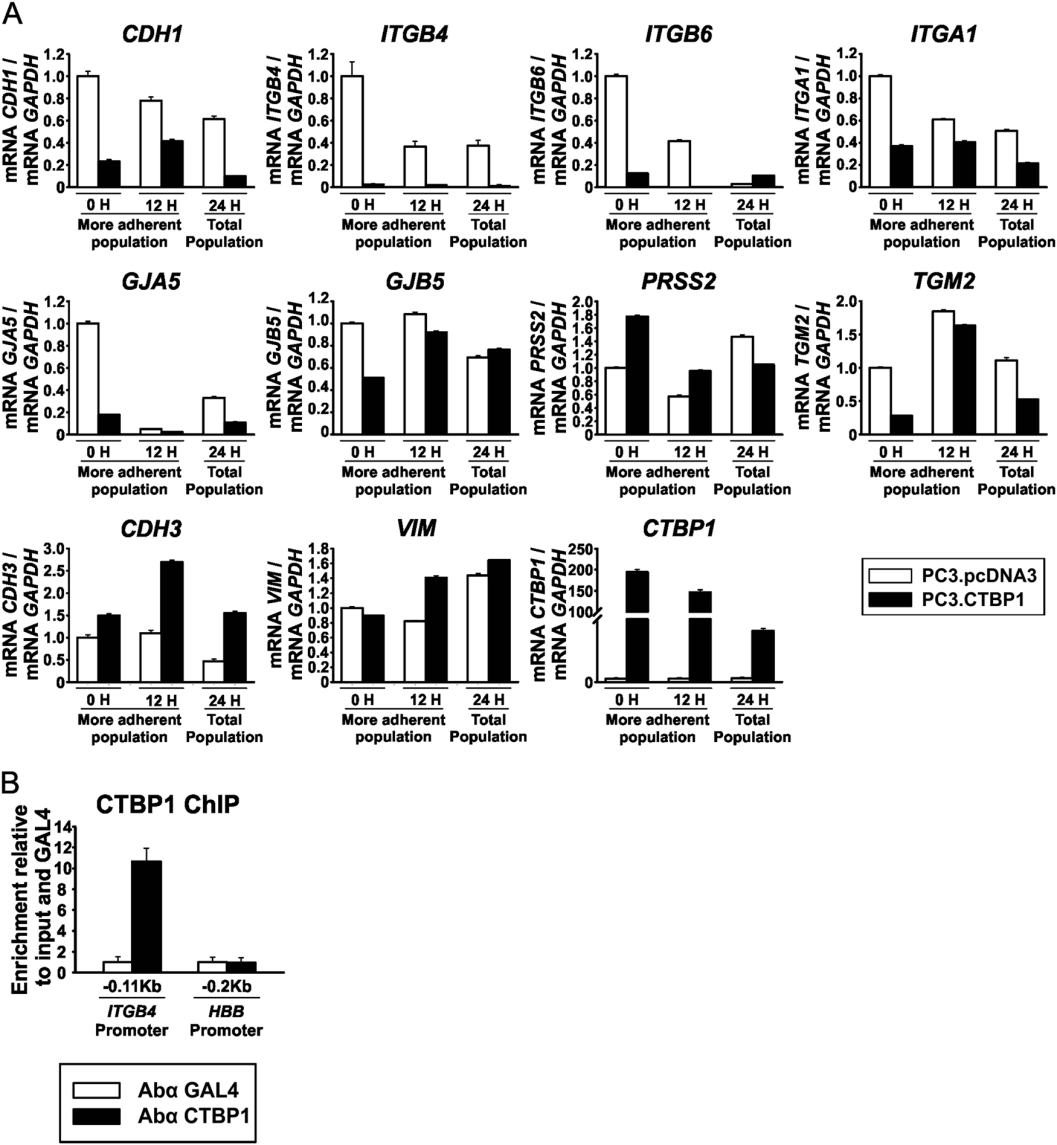
CTBP1 modulates the expression of cell adhesion genes and favors a mesenchymal phenotype. Gene expression was assessed by RT-qPCR at different times, after seeding PC3 cells on a collagen matrix. **a** Expression levels of several cell adhesion genes were analyzed by RT-qPCR on total and adherent populations. Data were normalized to *GAPDH* and control. **b** ChIP-qPCR using CTBP1 or nonspecific (Gal4) antibodies and primers located at −0.11 kb upstream of the TSS of *ITGB4* or −0.2 kb upstream of the *HBB* TSS promoter region

In order to understand the molecular mechanism for CTBP1 role in cell adhesion, we studied gene-expression modulation by CTBP1 in different adherent cell populations. PC3 cells were transiently transfected to overexpress CTBP1. Twenty-four hours later cells were harvested and seeded on culture plates for 60 min. Culture medium was removed in order to eliminate the less adherent cell population and cells attached to the collagen matrix were harvested immediately or after 12 h. RNA isolation followed by RT-qPCR analysis from adherent and total cell populations demonstrated that CTBP1 transcriptionally repressed *CDH1*, *ITGB4*, *ITGB6*, *ITGA1*, *GJA5*, *GJB5*, *PRSS2*, and *TGM2* gene expression, while induced *CDH3* and *VIM* (Fig. 3a). Notably, *CTBP1* overexpression favored a mesenchymal phenotype since *CDH1*, an epithelial marker, was significantly repressed and *VIM*, a mesenchymal marker, was induced (Fig. 3a). These results are consistent with CTBP1 role modulating cell adhesion genes as was observed in the xenograft tumors.

Based on the strong repression of *ITGB4* by CTBP1, we explored CTBP1 binding to its promoter. Chromatin immunoprecipitation (ChIP) assay demonstrated that CTBP1 bound to *ITGB4* proximal promoter region (Fig. 3b).

### CTBP1 reduces cell membrane attachment to substrate and increases filopodia number

**Fig. 4:**
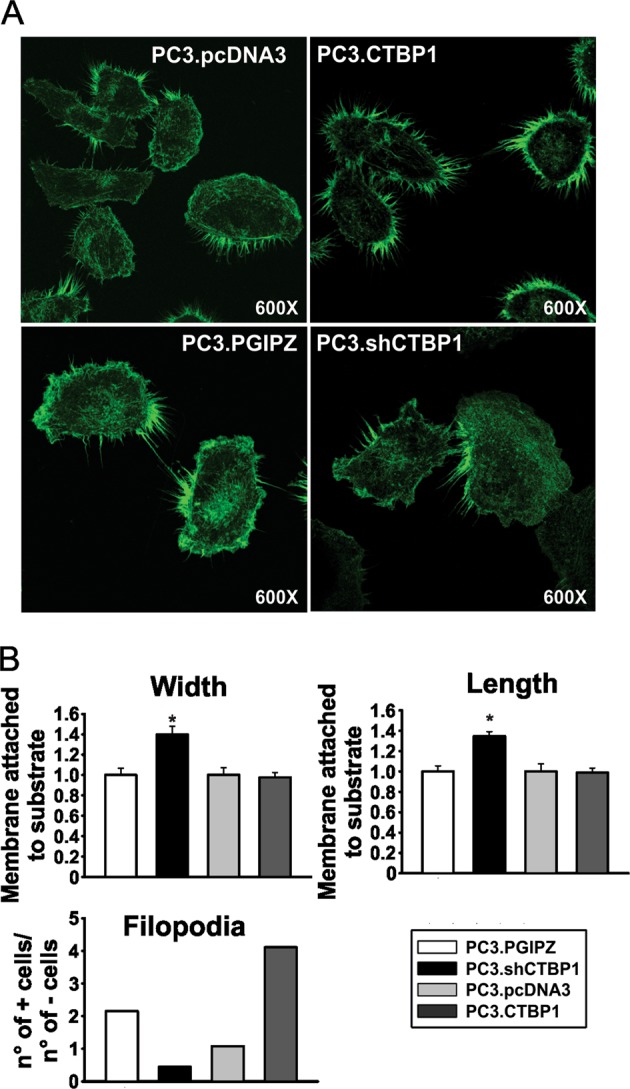
Analysis of the cell membrane portion involved in attachment to substrate. **a** Confocal microscopy images of rhodamine–phalloidin stained PC3 cells with differential CTBP1 expression levels. **b** Cellular dimensions, width and length, and the number of +cells (cells with a high count of filopodia) was estimated as cells with three or more filopodia sets. Cell dimensions were normalized to PC3.PGIPZ cells.PC3.PGIPZ, *n* = 23; PC3shCTBP1, *n* = 43; PC3.pcDNA3, *n* = 25; PC3.CTBP1, *n* = 35. Data were analyzed by one-way ANOVA. *p ≤ 0.05

To investigate the morphological alterations caused by CTBP1 regulation of cell adhesion, we explored the area of the cell membrane directly in contact with the substrate by rhodamine–phalloidin staining and confocal microscopy. No changes were found after CTBP1 overexpression; however, stable CTBP1 depletion lead to a higher amount of cell membrane attached to the substrate, observed by the increase in cell length and width (Fig. 4). Filopodia are thin, finger-like and highly dynamic actin-rich membrane protrusions that extend out from the cell edge and correlate with metastatic potential. Interestingly, CTBP1 overexpression increased the number of cells with a high count of filopodia (Fig. 4b). These results demonstrated that CTBP1 plays a role in cell–cell and cell-ECM (extra cellular matrix) adhesion molecules loss, the shift to a mesenchymal phenotype and pro-invasive cell morphology, suggesting the relevance of CTBP1 in the early stages of metastasis development.

### CTBP1 depletion impairs PC3 metastasis in HFD/MeS-like mice

**Fig. 5:**
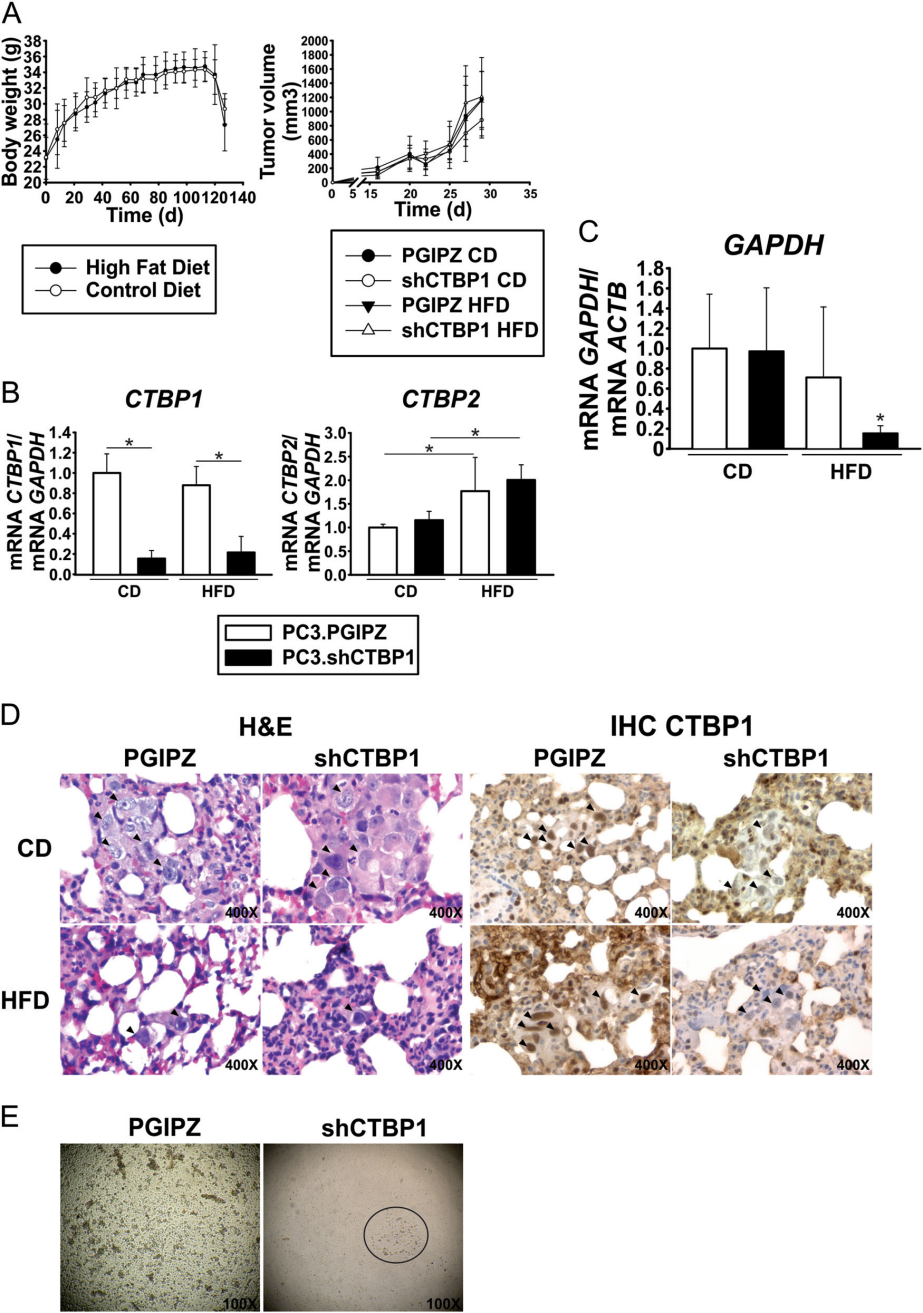
PCa and MeS spontaneous metastasis model. Four-week-old NSG mice were fed a CD or HFD for the entirety of the experiment. **a** Body weight was measured once a week (*left*) and on the 12th week of diet mice were inoculated in the left flank with PC3.PGIPZ (control) or PC3.shCTBP1 (CTBP1 depleted) cells. Tumor size was measured three times a week (*right*). **b** *CTBP1* and *CTBP2* mRNA levels at the time of sample collection was assessed by RT-qPCR on tumor samples. **c** Lung metastasis burden was inferred by the mRNA levels of human *GAPDH*, detected in RNA samples from mice lung tissue. **d** H&E (*left*) and CTBP1 IHC (inmunohistochemical) staining (*right*) from lungs of mice bearing xenografts, arrows indicate metastatic cells. **e** Representative images of CTCs harvested from PC3.PGIPZ (*n* = 1) or PC3.shCtBP1 (*n* = 3) inoculated NSG mice, 1 week after blood extraction. Bars represent average and SD of two independent experiments. Data were analyzed by two-way ANOVA. *p ≤ 0.05

To further investigate CTBP1 role in metastasis, we generated an in vivo MeS and PC3 metastasis model. NOD Scid Gamma (NSG) mice were fed with HFD or CD. On the 12th week CTBP1-depleted or control PC3 cells were inoculated on the animal left flank. Four weeks later, mice were sacrificed and tumor and lung samples were excised for RNA isolation, followed by RT-qPCR, and histopathological analysis. Regarding tumor growth and body weight, no differences were observed between treatments (Fig. 5a). However, a dramatic body weight loss beginning about 15 days after cell inoculation was observed (Fig. 5a), suggesting metastatic disease in these mice.

CTBP1 depletion at the end of the experiment was confirmed by RT-qPCR (Fig. 5b). Although, CTBP2 expression levels were not affected by CTBP1 depletion in xenografts, HFD significantly increased CTBP2 mRNA levels compared to CD-fed mice group (Fig. 5b).

During the autopsy procedure, no macrometastases were observed in the mice visceral organs. However, we detected *GAPDH* gene expression by RT-qPCR using specific human primers in RNA samples from lungs, as a first evidence of micrometastasis. As shown on Fig. 5c, CTBP1 depletion on HFD-fed mice resulted in lower-*GAPDH* expression compared to other groups which indicated less human cells in lungs and, in turn, less metastasis. Histopathological analysis was also performed by hematoxylin and eosin (H&E) staining confirming the metastasis reduction in CTBP1-depleted HFD-fed mice (Fig. 5d, Table 1). Furthermore, immunohistochemical (IHC) staining of CTBP1 showed that lung metastasis from CTBP1-depleted HFD mice had less CTBP1 protein compared to other mice groups (Fig. 5d).

**Table 1:** Histological analysis of lung metastases

		Lung metastases	
		SCORE	CTBP1
CD	PGIPZ	3.2	Positive
	shCtBP1	2.4	Negative
HFD	PGIPZ	3	Positive
	shCtBP1	1	Negative

NSG mice fed with HFD or CD were inoculated with PC3.PGIPZ or PC3.shCTBP1 cells. Lung metastasis score was determined based on metastatic focus number and size (cell number) by H&E staining.

Based on CTBP1 role in PCa cell adhesion and EMT, we also recovered circulating tumor cells (CTCs) from the peripheral blood of these mice. The colony formation capabilities of the CTCs were evaluated in a clonogenic assay. We found that CTBP1 depletion dramatically decreased the percentage of mice with CTC foci compared to PGIPZ control mice (Fig. 5e).

### PCa metastasis show high *VIM* and low-*CDH1* and *ITGB4* expression compared to primary tumors

**Fig. 6:**
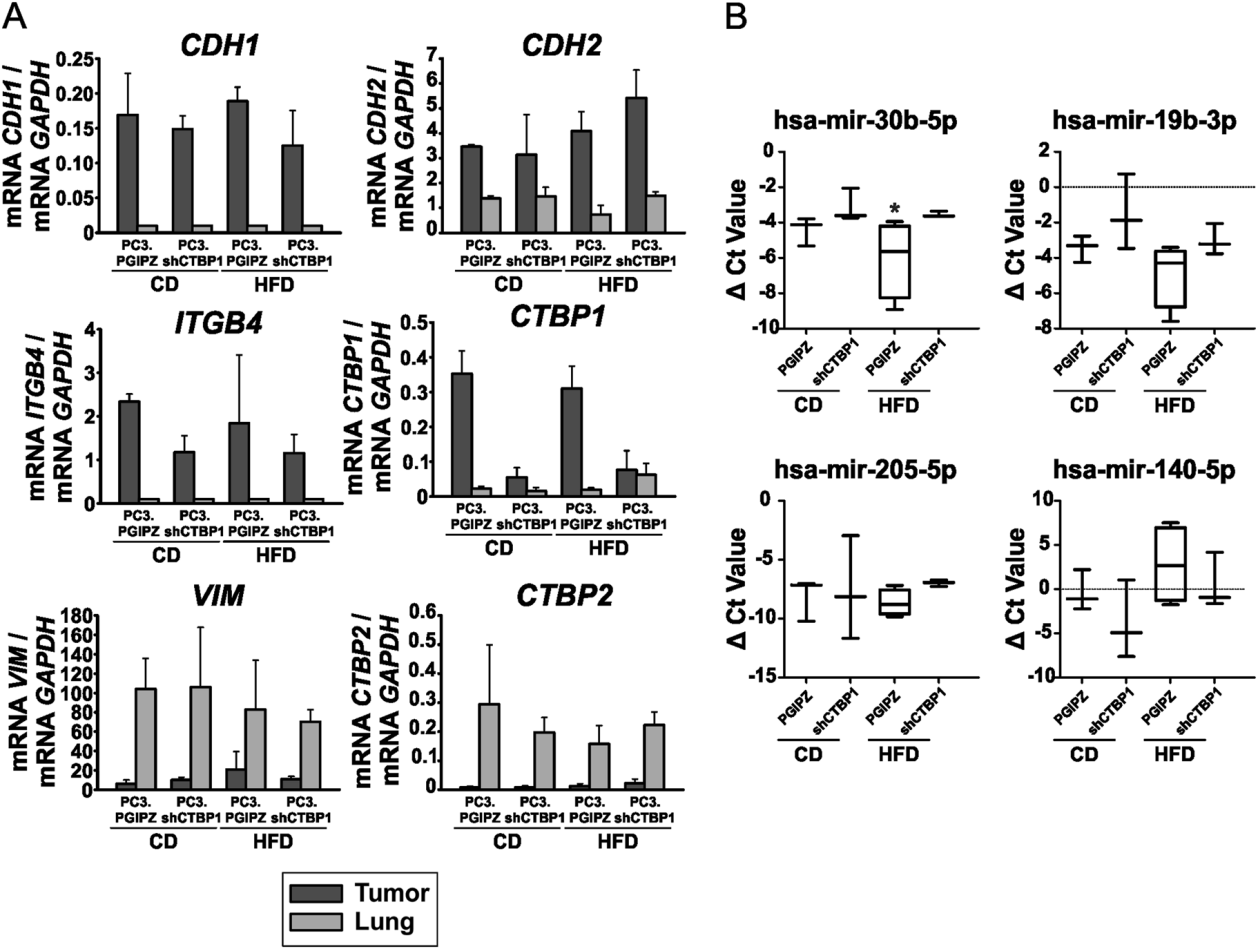
Expression levels of relevant genes between localized tumor cells and metastatic cells. Circulating miRNAs in mice plasma. **a** Key genes involved in EMT–MET were analyzed by RT-qPCR between tumor and lung samples. *CDH1* and *ITGB4* expression levels were not detected on metastasis samples. Bars represent average and SD of a representative experiment. **b** Plasma samples were analyzed by RT-qPCR for the indicated miRNAs and normalized to spike in cel-39 synthetic miRNA (ΔCt value = Ct_cel-39_ − Ct_sample_). Data were analyzed by *t* test. *p ≤ 0.05. Three mice were tested per experimental group

We compared the expression levels of key genes between primary tumors and human metastatic cells in the lung (Fig. 6). We found that metastatic cells had nearly null expression of the epithelial marker *CDH1* and the cell–ECM adhesion molecule *ITGB4*, expressed significantly higher levels of *VIM* and *CTBP2* and lower levels of *CTBP1*. However, PC3.shCTBP1 cells from lungs of HFD-fed mice, expressed similar levels of *CTBP1* mRNA to those of the primary tumors (Fig. 6). Histological and RT-qPCR analysis of the primary tumors did not show differences between CD and HFD (data not shown).

### CTBP1 and HFD reduces hsa-mir-30b-5p plasma levels in mice

We next tested the miRNA panel from Fig. 1b on mice plasma samples to detect circulating miRNAs in mice fed with CD or HFD and injected with CTBP1-depleted PC3 cells or control. After miRNA RT-qPCR, we only detected hsa-mir-30b-5p, hsa-mir-19b-3p, hsa-mir-205-5p and hsa-mir-140-5p (Fig. 6b). Interestingly, hsa-mir-30b was significantly decreased in plasma from HFD-fed mice (Fig. 6b). All the remaining miRNAs from Fig. 1b tested (hsa-miR-454-3p, hsa-miR-301a-3p, hsa-miR-29c-3p, hsa-miR-184, hsa-miR-335-5p, and hsa-miR-6799-3p) were undetected in mice plasma. These results show for the first time that low-circulating miR-30b-5p correlates with high metastasis suggesting that this miRNA might have a role triggering metastasis in HFD-fed animals.

## Discussion

The results presented on this work manifest the important role of CTBP1 and MeS on PCa progression and metastasis development. Metastasis is the major responsible of PCa mortality, and MeS has become a predominant risk factor for its incidence and progression^2,9^. In the present work, we presented new evidence linking both phenomena in CTBP1 and the plethora of genes it regulates either, directly or through miRNAs.

The role of CTBP1 in EMT and metastasis has been stated before in hepatocellular carcinoma^20^ and breast cancer^18,21^. More importantly, Wang et al.^22^ reported that mice injected through the tail vein with CTBP1-depleted PC3 cells had less metastasis development compared to control. Our results reinforced these findings using an in vivo model for cancer progression that recapitulates the metastatic process from the initial steps of EMT: cell detachment, invasion, and extravasation. Hence, we report for the first time that CTBP1 together with MeS play a crucial role inducing PCa progression from localized to metastatic disease.

Concerning EMT initiation, we showed that CTBP1 overexpression diminished PCa cell adhesion regardless of androgen sensitivity. This effect is most likely the result of CTBP1 repression of cell adhesion molecules, such as cadherins, connexins, and integrins. In the case of LNCaP cells, the effect of CTBP1 was subtle. Considering that these cells have higher-CTBP1 expression compared to the other PCa cell lines^14^, it might be that adhesion is at its lowest and could not be affected by CTBP1 overexpression.

In addition, we found that CTBP1 increases the number of filopodia favoring a pro-invasive phenotype. Accordingly, CTBP1 depletion lead to a higher proportion of cell surface attached to substrate and the derepression of cell adhesion genes in xenograft tumors.

Many of the genes regulated by CTBP1 and related to cell adhesion were novel targets with unknown role in PCa. Nonetheless we found some reports regarding their role in other types of cancer and diseases. As an example, COL17A1 is a transmembrane protein of the collagen family and a structural component of hemidesmosomes. It has been reported that COL17A1 promotes cell adhesion, which is consistent with our findings, but also increases cell motility and migration in wound healing process and in the leading front of some invasive carcinomas^23,24^.

Considering these results, we explored CTBP1 effects on a mice model of MeS and spontaneous PCa metastasis. Remarkably, CTBP1 depletion impaired the development of lung metastases only on HDF fed mice.

Another important aspect is that *CTBP2* expression was not altered as a consequence of CTBP1 depletion. This is not minor since CTBP1 and 2 share some overlapping functions and because one of the ways CTBP1 enters the nucleus is by forming a herterodimer with CTBP2^25^. However, CTBP1 and 2 might have different roles in metastasis progression, since *CTBP2* expression is higher in metastatic cells compared to localized tumors, while oppositely CTBP1 expression is low in metastasis. This might indicate that CTBP1 distinct functions from CTBP2 could be more relevant in the initial steps of metastasis, mainly loss of cellular adhesion and invasion increase, while CTBP2 would gain importance in the newly established metastatic niche.

In addition, we reported a miRNA cluster associated to cell adhesion regulated by CTBP1. These miRNAs are downregulated in CTBP1-depleted cells, which would be expected of miRNAs whose targets are upregulated in the same conditions. In the future this miRNA cluster might be validated in patient samples with the ultimate goal of identifying potential biomarkers for PCa progression associated to MeS.

In particular, miR-205-5p was upregulated in CTBP1-depleted cells. This miRNA shows much promise as a therapeutic tool since it has been reported as an antimetastatic miRNA for its role in EMT inhibition through ZEB1 silencing^26^. It is possible that an EMT regulatory loop which includes ZEB1, CTBP1, and miR-205-5p may exist. As cancer advances in a MeS context, CTBP1 expression and/or activity would increase, leading to the repression of miR-205-5p and the upregulation of ZEB1, kick-starting the EMT process.

The role of miRNAs in PCa has been reviewed previously by our group^12^. miRNAs are involved in different aspect of PCa progression from the acquisition of a CRPC phenotype, like the miR 221/222 cluster^27^, to the promotion of EMT and the development of metastases as is the case of miR-22 role in repressing the epithelial marker CDH1 promoting cell invasion and migration^28^. Regarding the role of miR-30b-5p, the literature shows that this miRNA is cancer type dependent. Some authors show that miR-30b overexpression correlates with stage, metastatic potential, shorter time to recurrence and reduced overall survival on melanoma patients, and that ectopic overexpression of miR-30b/30d promotes a metastatic behavior in melanoma cells^29^. Other authors show that overexpression of miR-30b promotes apoptosis and suppresses tumor migration and invasion in gastric cancer cell lines^30^.

Regarding the genes regulated by CTBP1, it was not reported TGM2, COL17A1, GJB5, GJA5, ITGA1, and ITGB6 roles in PCa. However, oppositely to our findings, it was published that ITGB4^31,32^ and LCN2^33,34^ overexpression increased migration of PCa cell lines. Our findings characterize for the first time these genes function in vivo. Thus, the heterogeneity of cancer might explain the apparent different role for ITGB4 and LCN2 in PCa.

**Fig. 7:**
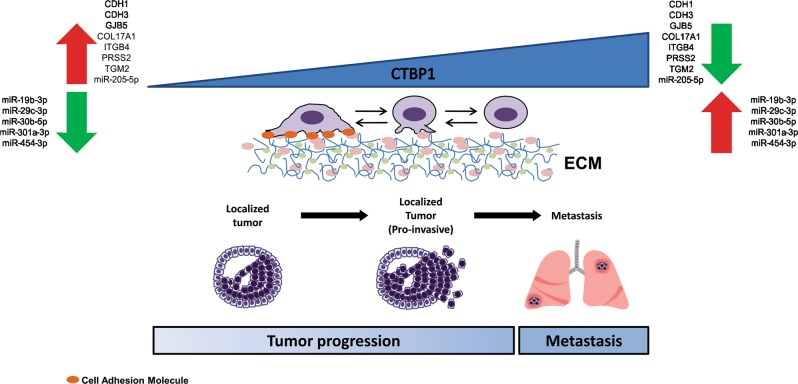
Hypothetical model. In early stages of PCa, localized tumor cells are highly adhesive, mostly epithelial and noninvasive, making the development of metastasis difficult. In this scenario, expression of CTBP1 is low hence cellular adhesion molecules and metastasis suppressor miRNAs are highly expressed while oncomiRs are repressed. As PCa progresses CTBP1 expression will increase and, in the case of MeS, its activity will also be enhanced. This will result in the repression of cell adhesion molecules and miR-205-5p together with upregulation of oncomiRs and changes in the cytoskeleton culminating in a less cohesive, mesenchymal oriented, and pro-invasive tumor prone to metastasis

In light of our evidence (see Fig. 7 for hypothetical model) we propose that, as PCa advances in a MeS context, CTBP1 activity and/or expression will increase, resulting in the differential regulation of mRNAs and miRNAs. The repression of cell adhesion molecules and miR-205-5p together with upregulation of oncomiRs and changes in the cytoskeleton will result in a less cohesive, mesenchymal oriented and pro-invasive tumor prone to metastasis. Altogether, our findings explain for the first time why MeS induces PCa progression identifying CTBP1 as the crucial player. Further research it's necessary to prove CTBP1 as a candidate for PCa treatment in MeS patients.

## Materials and methods

### Cell culture, plasmids, transfections, and treatments

PC3 (ATCC: CRL-1435), 22Rv1 (ATCC: CRL-2505), DU145 (ATCC: HTB-81), LNCaP (ATCC: CRL-1740), and C4-2^35^ cell lines and its stable derivatives were grown in RPMI 1640 (Invitrogen) supplemented with 10% of fetal bovine serum and antibiotics in a 5% CO_2_ humidified atmosphere at 37 °C. These cell lines were recently validated at MDA Cancer center (Texas, USA). PC3.shCTBP1 and its control (PC3.PGIPZ) stable cell lines were previously described^14^.

PC3.pcDNA3, pcDNA3.CTBP1 and PC3.ZEB1 cells were generated by transient transfection using 6 μg of plasmid and polyethylenimine methodology (PEI—PolySciences Inc.) with PEI:DNA ratio 2:1. CTBP1 plasmid and its control (pcDNA3) were previously described^17^. Zinc Finger E-Box Binding Homeobox 1 (ZEB1) plasmid was previously reported^36^.

### RNA isolation, cDNA synthesis, and qPCR (RT-qPCR)

Total RNA from cells, tumor xenografts, lungs, or plasma was isolated using TriReagent (Molecular Research Center). cDNA synthesis was performed using 2 µg of RNA and RevertAid First Strand kit (Fermentas, Vilnius, Lithuania). Real-time PCR (qPCR) was performed using TAQ Pegasus (Productos Bio-Lógicos, Argentina) in a CFX96 Touch™ Real-Time PCR Detection System (Bio-Rad). Data were normalized to actin beta (*ACTB)* or glyceraldehyde-3-phosphate dehydrogenase (*GAPDH*) and control. Primer sequences are shown in Supplemental Table 1.

### miRNA retrotranscription and qPCR (miRNA RT-qPCR)

miRNAs were retrotranscribed using the stem–loop method as previously described^37^ with some modifications. Briefly, 100 ng of total RNA were preheated at 70 °C during 5 min in 14 μL containing 0.07 μM of stem–loop primer. For plasma samples, cel-39 synthetic miRNA was spiked in before RNA extraction, and 4 μl of total RNA were used for retrotranscription. Retrotranscription was performed using M-MLV reverse transcriptase (Promega) and incubated in MyGenie96 Thermal Block (Bioneer) for 30 min at 16 °C, 50 min at 37 °C and 15 min at 70 °C. qPCR was performed in 25 μL with 0.05–1 μL RT product, 1U Taq DNA polymerase (Pegasus), 4 mM MgCl_2_, 0.2 mM dNTPs, 3 × 10^−5^ μL Sybrgreen (Sigma), 0.1 μM forward primer, and 0.1 μM reverse primer. The reactions were incubated in StepOne Plus Real Time PCR (Applied Biosystems) at 94 °C for 2 min, followed by 40 cycles of 95 °C for 15 s, annealing temperature for 20 s and 72 °C for 25 s. All reactions were run in duplicate. The expression levels of miRNAs were normalized to hsa-miR-103a-3p levels and control. Primer sequences for miRNA RT-qPCR are listed in Supplemental Table 1.

### Chromatin immunoprecipitation

ChIP was performed from PC3 cells using specific antibodies for CTBP1 (Santa Cruz Biotechnologies) or nonspecific Gal4 antibody (Santa Cruz Biotechnologies), as previously described^14^. ChIP-DNA was amplified by qPCR using primers located at −0.11 and 0 kb upstream of the TSS of *ITGB4* or −0.2 kb upstream of the hemoglobin subunit beta (*HBB*) TSS (Supplemental Table 1). Fold enrichment was calculated normalizing data to input and Gal4.

### Cell adhesion assay

Cell adhesion assay was performed as previously described with some modifications^38^. Briefly, cells were transfected as indicated and then seeded on a 96-wells culture plate, with or without a collagen coating, followed by 30 or 60 min of incubation. Afterward culture media was removed and cells were washed twice with phosphate-buffered saline, fixed with methanol and stained with crystal violet (CV) for 10 min. Excess CV was washed with distillated water twice. Stain dissolved in a solution 10% methanol: 5% acetic acid was determined with enzyme linked immunosorbent assay Multiskan FC (Thermo Scientific) absorbance at 620 nm.

### Immunofluorescence experiments and confocal microscopy

PC3 cells (transfected or otherwise) were fixed with 8% paraformaldehyde (20 min, room temperature) and stained with rhodamine–phalloidin (1 h, room temperature). Confocal images were acquired by confocal microscopy (FV1000, Olympus, Tokyo, Japan) using an UPlanSApo 60× oil immersion objective (NA 1/41.35; Olympus), a diode laser of 543 nm as the excitation source and fluorescence was collected in the range of 555–655 nm. We selected the regions closest to the substrate from which filopodia were clearly defined.

Confocal microscope images were analyzed using ImageJ software (NIH, Bethesda, MD, USA). Cells were considered positive (high-filopodia count) if they presented more than three or more filopodia sets.

### MeS and PCa spontaneous metastasis model

Four-week-old male NOD Scid Gamma (NSG) mice (*N* = 20), were housed under pathogen free conditions following the IBYME’s animal care guidelines. Mice were randomized into 2 dietary groups and fed ad libitum during 16 weeks with control diet (CD; 4640 kcal/kg, 5% fat) or HFD (6,040 kcal/kg, 37% fat). Body weight was monitored once a week. After 12 weeks of diet, mice on each dietary group were randomly distributed into 2 groups and injected s.c with PC3.PGIPZ or PC3.shCTBP1 cells (4.8 × 10^6^ cells per mouse). Tumor volume was determined three times a week and calculated as previously described^39^. Animals were sacrificed in the 16th week and tumor, liver, lung, and blood samples were collected.

### Histological and IHC analysis

Tissue samples collected from PCa and MeS mice described above were formalin-fixed and paraffin embedded. For histological analysis, 4 μm microscopic sections were stained with hematoxylin-eosin (H&E) and examined by light microscopy. For IHC analysis of CTBP1, anti-CTBP1 antibody (1:400; BD Biosciences) was used. The procedure was completed using a streptavidin–biotin–complex method (VECTASTAIN^®^  Universal Elite^®^ ABC Kit, Vector Laboratories, Maravai LifeSciences) with 3,3′ diaminobenzidine (DAB) as chromogen and examined by light microscopy. IHC evaluation was performed by a pathologist without knowledge of the grouping information.

### CTCs assay

To recover CTCs from peripheral blood, plasma samples (extracted from HFD-fed NSG mice at the time of sacrifice) were treated with ammonium chloride potassium buffer (1:1; 3 × 7 min; 1 × 5 min) in order to lysate erythrocytes and then centrifuge. The cell pellet was resuspended on fresh complete RPMI medium supplemented with puromycin (1 μg/ml) and seeded on a 96-wells cell plate. One-week later, cells were photographed with a Q-Color5 Digital Camera (OLYMPUS).

### Statistical analysis

All results are given as mean and standard deviation of three independent experiments unless stated otherwise. Student's *t* tests or two-way ANOVA followed by Tukey test were performed. Shapiro–Wilk and Levene tests were used to assess normality and homogeneity of variances. *P < 0.05; **P < 0.01; ***P < 0.001.

## Supplementary information

Supplemental table
